# High frequency switched-mode stimulation can evoke post synaptic responses in cerebellar principal neurons

**DOI:** 10.3389/fneng.2015.00002

**Published:** 2015-03-06

**Authors:** Marijn N. van Dongen, Freek E. Hoebeek, S. K. E. Koekkoek, Chris I. De Zeeuw, Wouter A. Serdijn

**Affiliations:** ^1^Section Bioelectronics, Faculty of Electrical Engineering, Mathematics and Computer Science, Delft University of TechnologyDelft, Netherlands; ^2^Department of Neuroscience, Erasmus Medical Center RotterdamRotterdam, Netherlands; ^3^Netherlands Institute for Neuroscience, Royal Dutch Academy of Art and ScienceAmsterdam, Netherlands

**Keywords:** computational neuroscience, pulsatile stimulation, switched-mode operation, Purkinje cells, patch-clamp recordings

## Abstract

This paper investigates the efficacy of high frequency switched-mode neural stimulation. Instead of using a constant stimulation amplitude, the stimulus is switched on and off repeatedly with a high frequency (up to 100 kHz) duty cycled signal. By means of tissue modeling that includes the dynamic properties of both the tissue material as well as the axon membrane, it is first shown that switched-mode stimulation depolarizes the cell membrane in a similar way as classical constant amplitude stimulation. These findings are subsequently verified using *in vitro* experiments in which the response of a Purkinje cell is measured due to a stimulation signal in the molecular layer of the cerebellum of a mouse. For this purpose a stimulator circuit is developed that is able to produce a monophasic high frequency switched-mode stimulation signal. The results confirm the modeling by showing that switched-mode stimulation is able to induce similar responses in the Purkinje cell as classical stimulation using a constant current source. This conclusion opens up possibilities for novel stimulation designs that can improve the performance of the stimulator circuitry. Care has to be taken to avoid losses in the system due to the higher operating frequency.

## 1. Introduction

Traditional functional electrical stimulation typically uses a current source with constant amplitude *I*_*stim*_ and pulsewidth *t*_*pulse*_ to recruit neurons in the target area. Early stimulator designs consisted of relatively simple programmable current source implementations. Over the years numerous modifications have been proposed to improve important aspects such as power efficiency (Sooksood et al., 2012), safety (Sooksood et al., 2010) and size. Most stimulators however, still use constant current at the output.

Several studies have investigated the use of alternative stimulation waveforms in an attempt to improve the performance. Some implementations focus on improving the efficiency of the activation mechanism in the neural tissue. In Sahin and Tie (2007) and Wongsarnpigoon and Grill (2010) it was found that Gaussian shaped waveforms increase the neural recruitment efficiency as compared to standard rectangular pulses. In Hofmann et al. (2011) it was found that the efficiency increases by introducing an inter-pulse delay in a biphasic stimulation scheme. Other implementations employ alternative waveforms to improve the performance of the stimulator circuit. In van Dongen and Serdijn (in press) we proposed to replace the rectangular constant current with a high-frequency pulse train. In this implementation a stimulation signal is composed of many very short current spikes.

The advantage of this high frequency pulsed approach is that it can improve the power efficiency of the stimulator circuits. Traditional constant current stimulators deliver current from a fixed supply voltage. When only part of this supply voltage is used during stimulation, the power efficiency of the stimulator is rather low. In the proposed methodology the current pulses are not drawn from a fixed supply voltage, but are instead generated by repetitively discharging a charged inductor into the target tissue. This eliminates the wasted voltage headroom and can therefore improve the power efficiency. A prototype stimulator system has shown that efficiency improvements up 200% are possible as compared to state-of-the-art conventional stimulator designs. A higher power efficiency means that the size of the battery can be reduced, which is an important advantage for implantable stimulator systems.

Another advantage of the high frequency stimulator is coming from the possibility to steer the high frequency current pulses to various electrodes in an alternated fashion. By adjusting the strength of the individual current pulses, it is possible to send tailored stimulation patterns to multiple electrodes at the same time. This makes the technique very suitable for multi-electrode stimulation configurations, such as encountered in field steering applications as described in e.g., Martens et al. (2011) and Valente et al. (2012). Since a single stimulator circuit can target many electrodes independently, it offers more flexibility as compared to conventional stimulator systems.

The technical functionality of the proposed stimulator and the advantages described above have already been validated in van Dongen and Serdijn (in press). Instead, the current study answers the question whether the proposed novel high frequency stimulation signal can evoke a neural response in a similar fashion as during classical constant current stimulation. The electrophysiological feasibility of the new high-frequency pulsed excitation is investigated. First the response of axons to a high-frequency stimulation pattern is analyzed by taking into account the dynamic properties of both the tissue material as well as the axons. Subsequently an *in vitro* measurement setup is used to verify the response of Purkinje cells to such a stimulation signal applied to neuronal afferents. By comparing the high-frequency response to a classical constant current response, the efficacy of the stimulation is determined.

## 2. Materials and methods

**Figure 1:**
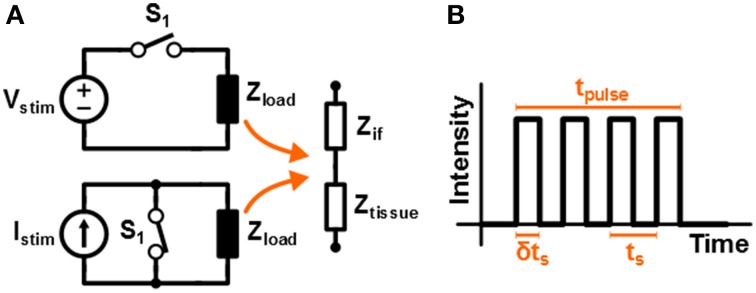
**(A)** Schematic representation of a high frequency voltage and current system that is driven by a switch that is controlled by a PWM signal. In **(B)** the resulting stimulation signal is sketched.

The high frequency stimulation pattern that is used in this work to stimulate the tissue is assumed to be square shaped. The schematic circuit diagrams of both voltage and current based stimulation are depicted in Figure 1A. A fixed value for *V*_*stim*_ or *I*_*stim*_ is used, while the stimulation intensity is controlled by driving the switch with a Pulse Width Modulated (PWM) signal; this is referred to as switched-mode operation. In Figure 1B a sketch is given of the monophasic stimulation pulse resulting from either of the circuits. The switch is operated with duty cycle δ and switching period *t*_*s*_ = 1/*f*_*s*_. This results in an average stimulation intensity *V*_*avg*_ = δ*V*_*stim*_ or *I*_*avg*_ = δ*I*_*stim*_ for voltage and current based stimulation, respectively.

It is important to note that in this work the term 'high frequency' refers to the frequency of the pulses that make up a single stimulation waveform. It does not refer to the repetition rate at which the stimulation cycles are repeated. For example, if the pulse frequency *f*_*s*_ = 100 kHz, a stimulation signal of *t*_*pulse*_ = 200 μs would consist of 20 pulses.

The effect of this high-frequency stimulation waveform is analyzed and compared with a classical constant amplitude waveform in two steps: first the response is analyzed using modeling that takes into account the impedance of the neural tissue as well as the properties of the axons. The results are subsequently verified using an *in vitro* experimental setup. Both methods are discussed separately here.

### 2.1. Modeling

A high frequency (switched) signal that is injected via the electrodes will be filtered by the tissue. First the tissue material properties influence the transient voltage over and current through the tissue. Subsequently the electric field in the tissue and the properties of the cell membrane will determine the transient shape of the membrane voltage, which is ultimately responsible for the actual activation or inhibition of the neurons. These two processes will be discussed separately.

#### 2.1.1. Material properties

In Figure 1A the tissue is modeled with an interface impedance *Z*_*if*_ and a tissue impedance *Z*_*tis*_. For current based stimulation *V*_*tis*_ = *I*_*stim*_*Z*_*tis*_ is independent of *Z*_*if*_. For voltage based stimulation *V*_*tis*_ = *V*_*stim*_ − *V*_*if*_ with *V*_*if*_ the voltage over *Z*_*if*_. In this study non polarizable Ag/AgCl electrodes will be used for which *Z*_*if*_ ≈ 0 and therefore *V*_*tis*_ ≈ *V*_*stim*_ (Merrill et al., 2005).

**Figure 2:**
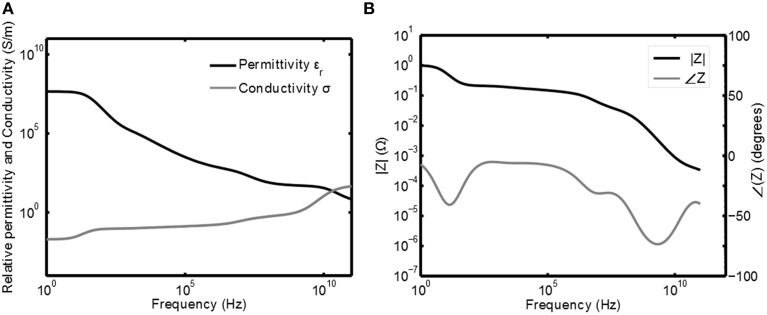
**Frequency response of gray matter**. In **(A)** the permittivity ϵ and conductivity σ are plotted as function of the frequency (Gabriel et al., 1996) and in **(B)** the corresponding normalized (impedance) Bode plots are given.

The tissue voltage *V*_*tis*_ and current *I*_*tis*_ are related to each other via the resistive and reactive properties of the tissue. In Gabriel et al. (1996) the capacitive and resistive properties of the tissue are measured for a wide range of frequencies and human tissue types. The resistivity and permittivity of gray matter as a function of the frequency are plotted in Figure 2A. This plot has been obtained by calculating the relative permittivity ϵ_*r*_ and conductivity σ based on the equation for the relative complex permittivity $\hat{\epsilon }$_*r*_(ω) from Gabriel et al. (1996):

(1) $$\epsilon_{r}(\omega )=\text{Re}\left[{\hat{\epsilon }}_{r}(j\omega )\right]$$

(2) $$\sigma (\omega )=\text{Im}\left[{\hat{\epsilon }}_{r}(j\omega )\right]\;\cdot \;-\;\epsilon_{0}\omega$$

Here ϵ_0_ is the permittivity of free space. As can be seen neural tissue shows strong dispersion for $\hat{\epsilon }$_*r*_(ω). To find the relation between the tissue voltage and current the values of ϵ_*r*_ and σ need to be converted to impedance. Given $\hat{\epsilon }$_*r*_ the impedance *Z* is:

(3) $$Z=\frac{1}{{\hat{\epsilon }}_{r}j\omega C_{0}}$$

Here *C*_0_ is a constant that sets the absolute value of the impedance, which depends among other things on the electrode geometry. It is possible to normalize the impedance, such that |*Z*(0)| = 1 by using:

(4) $$\begin{array}{l} \underset{\omega \to 0}{\lim}|Z(j\omega )|=\underset{\omega \to 0}{\lim}\;{\left[\sqrt{{\left(\epsilon_{r}(\omega )\right)}^{2}+{\left(\frac{-\sigma (\omega )}{\omega \epsilon_{0}}\right)}^{2}}\omega C_{o}\right]}^{-1}\\ \;=\frac{\epsilon_{0}}{\sigma (0)C_{0}} \end{array}$$

Here Equations (1, 2) are substituted for $\hat{\epsilon }$_*r*_(ω) and σ(0) is the conductance of the tissue at ω = 0. From this it follows that *C*_0_ = ϵ_0_/σ(0) to normalize the transfer such that |*Z*(0)| = 1. The Bode plots of this normalized impedance are given in Figure 2B. This plot can now be used to obtain the shape for *I*_*tis*_ and *V*_*tis*_ and, if the impedance of the tissue is known for a certain frequency, it can be scaled to obtain the correct absolute values.

**Figure 3:**
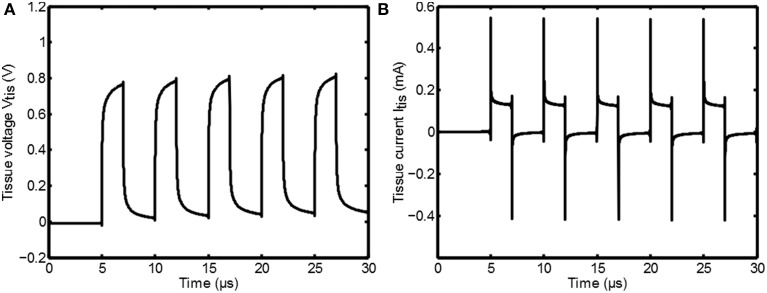
**The tissue voltage ***V***_*tis*_ resulting from a square wave current input **(A)** and the tissue current ***I***_*tis*_ resulting from a square wave voltage input **(B)**, based on the impedance as given in Figure 2B**.

As an example, a 100μ A, 200kHz, δ = 0.4 switched current signal *i*_*in*_(*t*) is supplied to an electrode system that has an impedance of |*Z*| = 10kΩ at 1kHz. The tissue voltage is now found by solving *V*_*out*_(*t*) = 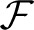
^− 1^[*Z* · 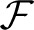
[*i*_*in*_(*t*)]], which is plotted in Figure 3A. Indeed the tissue voltage is filtered and in the next section it will be seen that this is important for determining the activation of the neurons.

Similarly a 1V, 200kHz, δ = 0.4 switched voltage signal *v*_*in*_(*t*) can be applied. The tissue current follows from *I*_*out*_(*t*) = 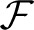
^−1^[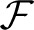
[*v*_*in*_(*t*)]/*Z*] and is plotted in Figure 3B. The current spikes in Figure 3B are due to the rapid charging of the capacitive properties of the tissue that arise from ϵ_*r*_(ω).

#### 2.1.2. Membrane properties

After the transient intensities of the tissue voltage and current have been determined by the stimulation protocol and the tissue impedance, it can be investigated how these quantities influence the neurons. Analogous to Warman et al. (1992) the activation of the neurons is considered in the axons, for which the membrane voltage is determined using the cable equations. For these equations first the potential in the tissue as a function of the distance from the electrode is needed. When the electrode is considered to behave as a point source at the origin, the tissue potential has a 1/*r* dependence assuming quasi-static conditions (Warman et al., 1992): Φ(*r*) = *I*_*stim*_/(σ4π *r*), where *r* is the distance from the electrode.

In Bossetti et al. (2008) the influence on the tissue potential due to high frequency components in the stimulation signal was analyzed. It was found that the propagation effect was negligible and that only the complex permittivity as discussed in the previous section was significant. To incorporate these properties, the potential Φ(*r*, *j*ω) can be determined in the frequency domain by substituting σ with the complex permittivity, leading to:

(5) $$\Phi (r,j\omega )=\frac{I_{tis}(j\omega )}{j\omega \epsilon_{0}{\hat{\epsilon }}_{r}4\pi r}.$$

By transforming this potential back to the time domain, the transient of the potential Φ(*r*, *t*) = 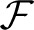
^−1^[Φ(*r*, *j*ω)] at any distance *r* from the point source is obtained. Since the current is divided by the complex permittivity and since there are no propagation effects, the transient shape of the potential is proportional to *V*_*tis*_ as obtained in Figure 3; it is just scaled as a function of the distance.

Next Φ(*r*, *t*) can be used as an input for an axon model to determine the response of the membrane voltage. The electrical parameters that are used for the axon model are summarized in Table 1 (taken from Warman et al., 1992 and Tai et al., 2005). In the following section the response of both myelinated and unmyelinated axons is considered. In both sections the fiber diameter is chosen to be small (*d*_*o*_ = 0.8μm), based on the granule cell axon diameter (Gray, 1961), which will be the target for stimulation during the *in vitro* experiments.

**Table 1:** **Axon properties used for the axon model (taken from Warman et al., 1992 and Tai et al., 2005)**.

**Symbol**	**Description**	**Value**
ρ_*i*_	Axoplasm resistivity	54.7Ω · cm
ρ_*o*_	Extracellular resistivity	0.3kΩ · cm
*c*_m_	Nodal membrane capacitance/unit area	2.5μF/cm^2^
ν	Nodal gap width	1.5μm
*l*/*d*_*o*_	Ratio of internode spacing to fiber diameter	100
*d*_*i*_/*d*_*o*_	Ratio of axon diameter to fiber diameter	0.6
*g*_*Na*_	Sodium conductance/unit area	120mS/cm^2^
*V*_*Na*_	Sodium reversal voltage	115mV
*g*_*K*_	Potassium conductance/unit area	36mS/cm^2^
*V*_*K*_	Potassium reversal voltage	−12mV
*g*_*L*_	Leakage conductance/unit area	0.3mS/cm^2^
*V*_*L*_	Leakage voltage	10.61mV

##### 2.1.2.1. Myelinated axons

For a myelinated axon the model as discussed in Warman et al. (1992) is used. At the nodes of Ranvier, the axon membrane is characterized by a membrane capacitance *C*_*m*_ = *c*_*m*_ π *d*_*i*_ ν in parallel with a series connection of a rest potential voltage source *V*_*rest*_ = − 70 mV and a nonlinear conductance *G*_*HH*_. The current through this conductance is given by the Hodgkin Huxley equations (Hodgkin and Huxley, 1952). The myelinated parts of the axon do not have ionic channels and instead connect the nodes of Ranvier by means of an intracellular resistance *R*_*i*_ = ρ_*i*_*l*/(π (*d*_*i*_/2)^2^, in which *l* represents the internode spacing and *d*_*i*_ the axon diameter.

The membrane voltage *V*_*m*, *n*_ at node *n* can be found by solving the following equation that follows directly from Kirchhoff's laws (Warman et al., 1992):

(6) $$\begin{array}{l} \frac{dV_{m,n}}{dt}=\frac{1}{C_{m}}[\frac{1}{R_{i}}(V_{m,n\;-\;1}-2V_{m,n}+V_{m,n\;+\;1}\\ \;+\;V_{o,n\;-\;1}-2V_{o,n}+V_{o,n\;+\;1})\;-\pi d_{i}\nu i_{HH}] \end{array}$$

Here *V*_*o*, *n*_ is the voltage due to the electric field at node *n* that follows from Equation (5) and *i*_*HH*_ is the current density given by the Hodgkin Huxley equations:

(7a) $$\begin{array}{l} i_{HH}=g_{Na}m^{3}h(V_{m,n}-V_{rest}-V_{Na})\\ \;+\;g_{K}n^{4}(V_{m,n}-V_{rest}-V_{K})\\ \;+\;g_{L}(V_{m,n}-V_{rest}-V_{L}) \end{array}$$

(7b) $$\frac{dm}{dt}=\alpha_{m}(1-m)-\beta_{m}m$$

(7c) $$\frac{dh}{dt}=\alpha_{h}(1-h)-\beta_{h}h$$

(7d) $$\frac{dn}{dt}=\alpha_{n}(1-n)-\beta_{n}n$$

The conductances *g*_*Na*_, *g*_*K*_, and *g*_*L*_ as well as the voltage *V*_*Na*_, *V*_*K*_, and *V*_*L*_ are constants, while α_*x*_ and β_*x*_ depend on the membrane voltage *V*′ = *V*_*m*_ − *V*_*rest*_ via:

(8a) $$\alpha_{m}=\frac{0.1\;\cdot \;(25-V^{\prime })}{\exp\frac{25-V^{\prime }}{10}-1}\;\alpha_{h}=\frac{0.07}{\exp\frac{V^{\prime }}{20}}\;\alpha_{n}=\frac{0.01(10-V^{\prime })}{\exp\frac{10-V^{\prime }}{10}-1}$$

(8b) $$\beta_{m}=\frac{4}{\exp\frac{V^{\prime }}{18}}\;\beta_{h}=\frac{1}{\exp\frac{30-V^{\prime }}{10}+1}\;\beta_{n}=\frac{0.125}{\exp\frac{V^{\prime }}{80}}$$

The response of the membrane potential due to the high frequency electric field can now be found by solving the differential equations above. This is done in Matlab by using the classical Runge-Kutta method (RK4). A step size of 1μs is chosen during the high frequency stimulation interval, while after the stimulation pulse a step size of 10μs is used. In Section 3 the response of the axon to a variety of switched-mode and constant current stimulation signals is shown.

##### 2.1.2.2. Unmyelinated axons

For unmyelinated axons the model is similar to the myelinated case: the axon is now divided into segments of length Δ*x* with each segment containing an intracellular resistance per unit length: *r*_*i*_ = 4ρ_*i*_/*d*_*i*_, the capacitance per unit area *c*_*m*_, the resting potential *V*_*rest*_ = −70 mV and the ionic conductance per unit area *g*_*HH*_. Again a differential equation can be found that solves the membrane voltage *V*_*m*, *n*_ (Rattay, 1986):

(9) $$\begin{array}{l} \frac{dV_{m,n}}{dt}=\frac{1}{c_{m}}[\frac{V_{m,n\;-\;1}-2V_{m,n}+V_{m,n\;+\;1}}{r_{i}{\left(\Delta x\right)}^{2}}\\ \;+\;\frac{V_{o,n-1}-2V_{o,n}+V_{o,n\;+\;1}}{r_{i}{\left(\Delta x\right)}^{2}}-i_{HH}] \end{array}$$

### 2.2. Experimental methods

To verify the results from the previous section, an *in vitro* experimental setup is used to compare the response of Purkinje cells to a high-frequency stimulation signal with the response to a classical constant current signal. In the following sections first the experimental setup and the recording protocol are discussed. Second, the circuit that is used to generate the high-frequency stimulation signal is described.

#### 2.2.1. Recording protocol

The *in vitro* recordings were performed in brain slices from the vermal cerebellum of C57Bl/6 inbred mice using a method similar to Gao et al. (2012). In short, mice were decapitated under isoflurance anesthesia and subsequently the cerebellum was removed and parasagittally sliced to preserve the Purkinje cell dendritic trees (250μm thickness) using a Leica vibratome (VT1000S). Slices were kept for at least 1 h in Artificial CerebroSpinal Fluid (ACSF) containing the following (in mM): 124 NaCl, 5 KCl, 1.25 Na_2_HPO_4_, 2MgSO_4_, 2CaCl_2_, 26 NaHCO and 20 d-glucose, bubbled with 95% O_2_, and 5% CO_2_ at 34°C. 0.1mM picrotoxin was added to the ACSF to block the inhibitory synaptic transmission from molecular layer inter-neurons. This approach allows recordings of excitatory post-synaptic responses in the Purkinje cells evoked by stimulation of granule cell axons.

Slices were kept in continuous presence of oxygenated ACSF (flow rate approximately 2.0ml/min at 32 ± 1°C). The Purkinje cells were visualized using an upright microscope (Axioskop 2 FS plus; Carl Zeiss) equipped with a 40x water-immersion objective.

The stimulus electrode is an Ag-AgCl electrode in a patch pipette pulled from borosilicate glass [outer diameter 1.65mm and inner diameter 1.1mm, World Precision Instruments (Sarasota, FL-USA)] and is filled with ACSF. This electrode has an impedance *Z*_*tis*_ ≈ 3MΩ and is stimulated using a monophasic cathodic stimulation protocol. The electrode is placed in the extracellular space of the molecular layer in the cerebellum lateral to where the dendritic tree of the Purkinje cells is assumed to be. We aimed to evoke neurotransmitter release from granule cell axons and to avoid direct depolarization of the Purkinje cell dendritic tree. Although we cannot ensure that we completely avoided this latter confounding factor, this commonly used experimental approach (see for instance, Zucker and Regehr, 2002; Belmeguenai and Hansel, 2005; Myoga and Regehr, 2011; Gao et al., 2012; Galliano et al., 2013) is sufficient to compare the activation mechanisms of the classical and high frequency stimulation waveforms. Note that direct depolarization of the Purkinje cell dendritic tree results in an obvious and detectable change in paired-pulse ratio, i.e., from paired-pulse facilitation (see **Figure 6**) to paired-pulse depression, much like that evoked by climbing fiber stimulation (not recorded in the current study; see Dittman and Regehr, 1998). Paired-pulse facilitation will be discussed further in the results section.

The response to the stimulus is recorded by whole cell patch-clamping Purkinje cells in the voltage-clamp mode using electrodes (same pipettes as the stimulus electrodes) filled with (in mM): 120 K-Gluconate, 9 KCl, 10 KOH, 3.48 MgCl_2_, 4 NaCl, 10 HEPES, 4 Na_2_ATP, 0.4 Na_3_GTP, and 17.5 sucrose, pH 7.25. The membrane voltage is kept at −65 mV with a holding current smaller than −500 pA (recorded using an EPC 10 double patch clamp amplifier and Pulse 8.80 software, HEKA electronics).

Two different kinds of stimulation are performed and the responses of the Purkinje cell are compared to each other. First of all classical stimulation is applied using a monophasic constant current source. For this purpose a Cygnus Technology SIU90 isolated current source is used. The amplitude of the current is varied to see the effect of stimulation intensity on the response of the Purkinje cell. The stimulation protocol consisted of two consecutive stimulation pulses with a duration of *t*_*pulse*_ = 700μs each and an interpulse interval of 25ms.

Second, switched-mode stimulation is performed, also using two pulses with *t*_*pulse*_ = 700μs and an interpulse interval of 25ms. If the Purkinje cell shows a similar response for varying δ during switched mode as it does for varying amplitude during classical stimulation, it can be concluded that switched-mode stimulation is indeed able to mimic classical stimulation.

#### 2.2.2. Stimulator design

**Figure 4:**
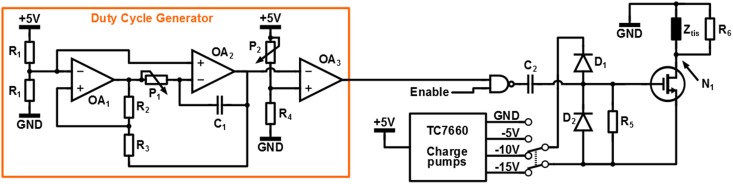
**Circuit used to generate a switched voltage monophasic stimulation protocol**.

The circuit used for switched-mode stimulation is depicted in Figure 4. As can be seen a switched-voltage stimulation scheme is applied: transistor *M*_1_ connects the electrode to the stimulation voltage *V*_*stim*_ = − 15V, *V*_*stim*_ = − 10V or *V*_*stim*_ = − 5V and is switched with a PWM signal of which the duty cycle δ determines the stimulation intensity.

The PWM signal is generated using the duty cycle generator circuit. Operational amplifiers (Opamps) *OA*_1_ and *OA*_2_ generate a triangular signal of which the frequency can be tuned using potentiometer *P*_1_. Subsequently the duty cycle δ is set using potentiometer *P*_2_ at the input of comparator *OA*_3_.

The circuit is controlled using an Arduino Uno micro-controller platform, which also supplies the circuit with a +5V supply voltage. The total circuit is isolated from ground by connecting the Arduino using the USB of a laptop that is operated from its battery. Capacitor *C*_2_ and clamps *D*_1_ and *D*_2_ are used to convert the 0-5V logic signal from the duty cycle generator to a *V*_*stim*_ to *V*_*stim*_ + 5V signal to drive the gate of *M*_1_. Resistor *R*_6_ = 1MΩ is used to discharge the gate of *M*_1_ to *V*_*stim*_ in steady state.

Because of the high electrode impedance, any parasitic capacitance that is connected to node *N*_1_ will prevent the electrode voltage to discharge during the 1 − δ interval of a switching period. This will influence the average voltage over the tissue and the relation between δ and the stimulation intensity. To prevent this effect, resistor *R*_5_ = 2.7kΩ is placed in parallel with the tissue, which allows the parasitic capacitance to discharge quickly. This resistor does consume power and reduces the power efficiency of the system dramatically. However, the power efficiency is not a design objective for this specific experiment: the only goal is to show the effectiveness of the high frequency stimulation. Without *R*_5_ the stimulation would still be effective, but the electrode voltage would not have the desired switched-mode shape. The whole circuit is implemented on a Printed Circuit Board (PCB).

## 3. Results

### 3.1. Modeling

**Figure 5:**
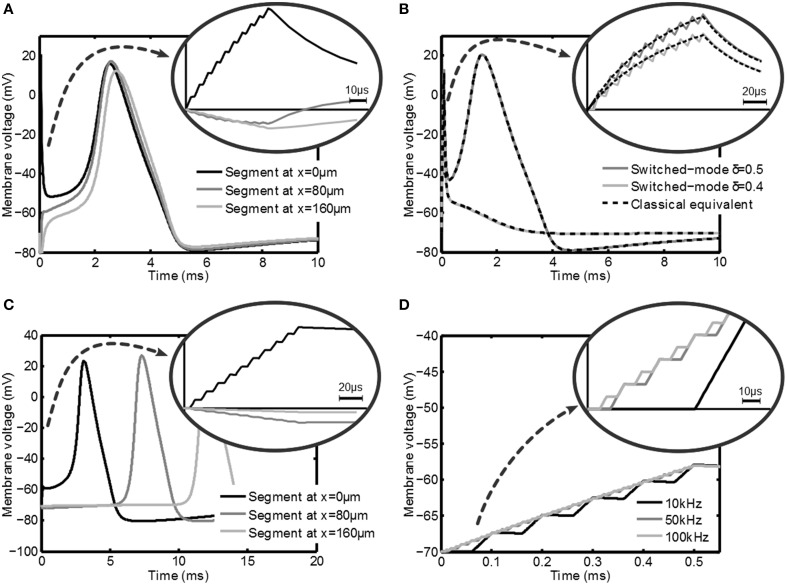
**Transient membrane voltage due to switched-mode stimulation for a variety of settings**. In **(A)** the membrane voltage at three nodes of Ranvier of a myelinated axon is depicted during and after stimulation with a δ = 0.5 switched-voltage source for which an action potential is generated. In **(B)** the effect of intensity (duty cycle for switched-mode vs. amplitude for classical stimulation) is depicted and compared. In **(C)** the response at three points in an unmyelinated axon is shown, where it is shown that it is also possible to create action potentials. In **(D)** the response of an unmyelinated axon is given for *f*_*s*_ = 10kHz, 50kHz and 100kHz (δ = 0.4), which shows that *f*_*s*_ has no significant influence on the activation. In all plots a zoom is given of the membrane voltage during the stimulation.

In this section the simulation results of the response of the axon to a variety of stimulation signals are discussed. First the response of a myelinated axon to a switched-voltage stimulation scheme is depicted in Figure 5A. In this figure *V*_*stim*_ = 1V, |*Z*(1kHz)| = 1kΩ, δ = 0.5, *f*_*s*_ = 100kHz and *t*_*pulse*_ = 100μs. An axon with the center node at a distance *y* = 0.5mm was considered. For this axon *C*_*m*_ = 56.6fF, *R*_*i*_ = 241.8MΩ, the nodes of Ranvier are spaced 80μm apart and a total of 9 nodes were simulated.

In Figure 5A the effect of the switched-mode stimulation can clearly be seen in the staircase transient shape of the membrane voltage. Furthermore, it can be seen that the increase in the membrane voltage also leads to an action potential in the axon. This shows that according to the models, switched-mode stimulation can induce activation in the axons. Finally, this action potential is able to travel along the axon, as is shown by the response of the other nodes of Ranvier in the same Figure. A very similar result can be obtained when using switched-current stimulation.

In Figure 5B the effect of the duty cycle δ is shown. The dark line shows the response for δ = 0.5 and the light line is the response for δ = 0.4. The latter setting is not able to induce an action potential, which shows that δ is an effective way of controlling the stimulation intensity. The response is compared with a classical constant voltage stimulation with *V*_*stim*, *classical*_ = δ*V*_*stim*_ and is indicated with the dashed lines. Indeed an equivalent response is found.

Next, the response of an unmyelinated axon is considered at a distance *y* = 0.5mm. The axon is divided into 501 segments of 1μm and has an outer diameter *d*_*o*_ = 0.8μm. For unmyelinated axons a higher stimulation intensity is needed in order to get effective stimulation. A voltage-mode stimulation signal with *V*_*stim*_ = 10V and δ = 0.5 is used. The membrane potential is depicted in Figure 5C and looks very similar to the myelinated response. Also in this case the action potential is able to travel along the axon as shown by the response of segments that are further down the axon. Note that the propagation speed is much lower than in the myelinated case for the standard Hodgkin-Huxley equations as used in the model. As will be seen from the experimental results, the actual propagation speed in the animal model will be higher.

Figure 5D shows the effect of varying *f*_*s*_: frequencies of 10, 50, and 100kHz are used. As can be seen both the membrane voltage after the stimulation pulse and the response of the tissue do not depend on *f*_*s*_.

### 3.2. Experimental results

**Figure 6:**
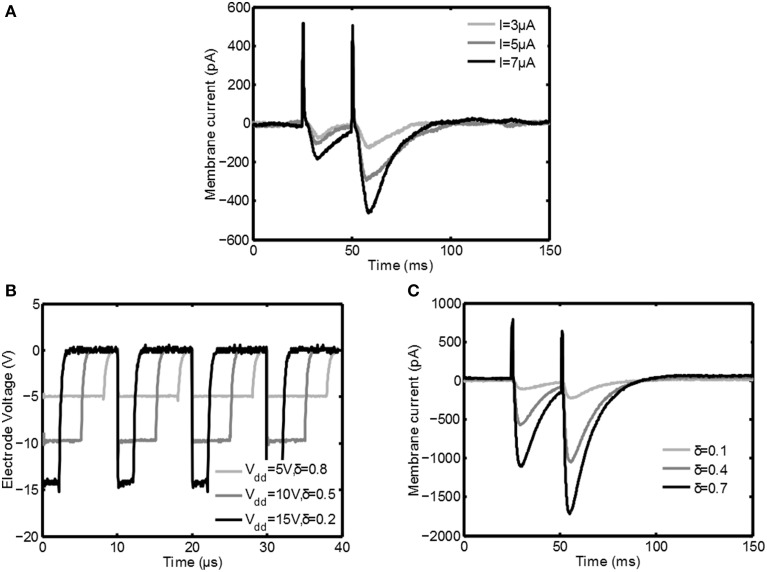
**Measurement results from the Purkinje cell during stimulation**. In **(A)** patchclamp recordings during classical constant current stimulation are depicted. In **(B)** the electrode voltage during switched-mode stimulation is plotted for various settings of *V*_*dd*_ and δ. In **(C)** the response of the neuron to switched-mode stimulation is shown. Both in **(A,C)** first a positive peak corresponding to the stimulation artifact is seen, after which an EPSC is generated that depends on the stimulation intensity.

In this section the measurements from the *in vitro* measurement setup are discussed. In Figure 6A the response of the Purkinje cell is shown for classical constant current stimulation for three different stimulus intensities. First there is a big positive spike corresponding to the stimulation artifact. After a small delay an excitatory postsynaptic current (EPSC) is clearly visible; during this interval the membrane current is decreased due to the opening of postsynaptic channels of the cell.

After 25ms the second stimulus arrives and a second EPSC is generated. This EPSC is much bigger due to a process called paired pulse facilitation (PPF): due to the first depolarization the Ca^2+^ concentration in the activated axon terminals is higher when the second pulse arrives, leading to an increased release of neurotransmitter (as reviewed by Zucker and Regehr, 2002). From the same figure it is also clear that the EPSC becomes stronger for increasing stimulation amplitude.

In Figure 6B the voltage over the stimulation electrode is plotted for various stimulation settings during switched-mode stimulation: both duty cycle δ as well as the supply voltage are varied with a fixed PWM frequency of 100kHz. Because of the voltage steered character the falling edge of the stimulation pulses is very sharp, while resistance *R*_5_ makes sure that it discharges reasonably fast.

In Figure 6C the response of the Purkinje cell is shown for switched-mode stimulation. For these plots *V*_*dd*_ = 15V, *t*_*pulse*_ = 700μs, and *f*_*s*_ = 100kHz. An EPSC with the same shape as during classical stimulation is the result and also the PPF is clearly visible. It is also seen that by increasing the intensity of the stimulation using δ the EPSC is increased, similar to how it is increased for classical stimulation using the stimulation amplitude. Furthermore, the average paired-pulse ratio (defined as the minimum of the second evoked EPSC divided by the minimum of the first EPSC) of the high frequency stimulation (mean 1.94, σ = 0.42) is very similar to the paired-pulse ratio for the constant current stimulation (mean 2.13, σ = 0.44) for comparable conditions. These points show that the switched-mode stimulation is able to induce similar activity in neural tissue as classical stimulation.

## 4. Discussion

The modeling shows that switched-mode stimulation is able to induce the same sort of activation as classical stimulation in both myelinated as well as unmyelinated axons. The duty cycle δ is used to control the stimulation intensity in exactly the same way as the amplitude for classical stimulation. Note that compared to the tissue material properties the membrane time constant is much larger and is therefore dominant.

**Figure 7:**
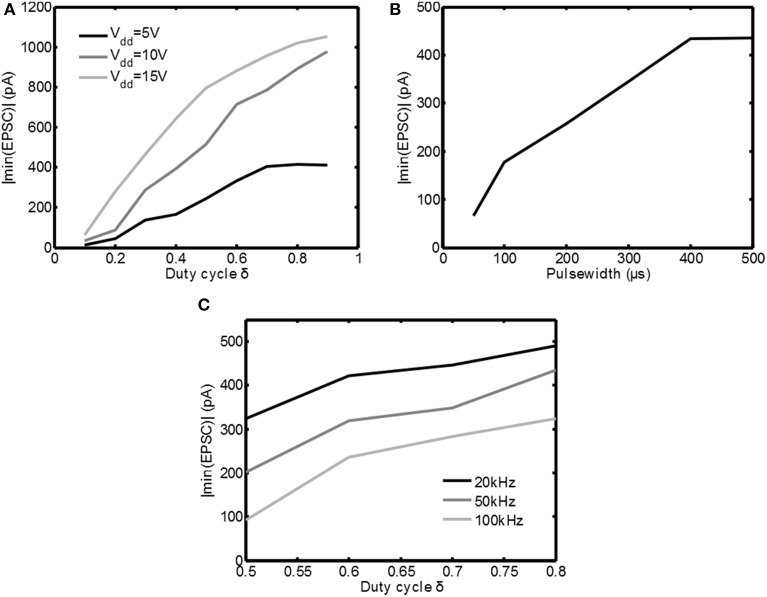
**Overview of the measurement results for various stimulation settings**. In **(A)** the absolute value of the minimum EPSC is plotted as a function of δ for various settings of *V*_*dd*_. In **(B)** the absolute value of the minimum EPSC is plotted as a function of the pulsewidth. In **(C)** the absolute value of the minimum EPSC is plotted for several PWM frequencies.

In Figure 7A the measurements are summarized by plotting the absolute value of the minimum in the EPSC | min(*EPSC*)| as function of the duty cycle δ(*f*_*s*_ = 100kHz, *t*_*pulse*_ = 250μs) for the three supply voltages available. Indeed for increasing supply voltage and/or increasing δ the response to the stimulation becomes stronger. This shows that both *V*_*dd*_ as well as δ are effective means of adjusting the stimulation intensity.

In Figure 7B the cell is stimulated with *V*_*dd*_ = 5V and δ = 0.6, while the pulse width was varied between 50μs and 500μs. As can be seen the minimum in the EPSC | min(*EPSC*)| is increasing for increasing pulse width, which is equivalent to the classical constant current stimulation. This confirms that also the pulse width is an effective means to adjust the stimulation intensity.

In Figure 7C the cell is stimulated with *V*_*dd*_ = 5V and *t*_*pulse*_ = 700μs, but the PWM frequency is varied from 20kHz up to 100kHz. As can be seen the stimulation intensity decreases for increasing frequency. This is an unexpected result, based on the simulations using the HH equations in Figure 5D. However, the simulations assumed that all the energy from the voltage source was transferred to *Z*_*tis*_. In reality this is not possible.

In Figure 3B large current peaks can be seen due to the charging of the capacitive component in *Z*_*tis*_. Any resistive component in series with *Z*_*tis*_ will reduce *V*_*tis*_ (the voltage over *Z*_*tis*_) during such a peak. Examples of these resistances could be a nonzero source impedance, the on resistance of the switch *M*_1_ and the faradaic interface resistance *Z*_*if*_ of the electrode. For increasing *f*_*s*_ = 1/*t*_*s*_ the amount of current peaks is increasing, which also increases the losses. This shows one of the disadvantages of using the switched-mode approach: losses can be expected due to the high frequency components in the stimulation waveform. Therefore, based on the measurement results, it can be concluded that switched-mode stimulation can lead to the same activation as classical stimulation, but care has to be taken to minimize additional losses that may arise due to the high frequency operation.

This paper didn't address the long-term effects of the switched-mode approach. The focus has been on comparing the short-term evoked high-frequency response with the classical response: the long-term response to high-frequency stimulation was not investigated. One of the long-term issues includes the consequences for tissue damage due to the use of the switched-mode approach. Most of the studies analyzing tissue damage (e.g., Shannon, 1992 and Butterwick et al., 2007) use a classic stimulation scheme only and therefore it is not known how their results translate to switched-mode operation. Another issue is that it is not proven that the evoked response to the high-frequency stimulation remains comparable to the classical stimulation over longer time.

Furthermore, the losses due to the high frequency operation are not quantified, since the stimulator circuit that was used did not allow for that. It would be required to compare the EPSC with the total amount of charge injected in the tissue (and not *R*_5_) during the stimulation pulse. Further investigation is needed to address these issues.

All in all this study confirms the electrophysiological feasibility for the design of stimulators that employ a high frequency output, although a trade-off needs to be made between the advantages that switched-mode operation can offer vs. the additional losses. Besides for the system proposed in van Dongen and Serdijn (in press), this conclusion has important implications for other stimulator systems as well. In Liu et al. (2008) a 250kHz pulsed waveform is used to decrease the size of the coupling capacitors. Two of these waveforms are added in anti-phase to reconstruct a conventional stimulation waveform. The conclusions from this paper show that it is not necessary to include the reconstruction step and instead the 250kHz waveform can be used to directly stimulate the tissue. This will lead to a further reduction in area consumption of the stimulator circuit.

Furthermore, in Arfin and Sarpeshkar (2012) a 10MHz forward-buck and reverse-boost converter is used to increase the power efficiency of the stimulator by using inductive energy recycling. External capacitors are used to low-pass filter the switched signal and reconstruct a conventional waveform. Also here the reconstruction step can be omitted and the switched signal can be used to stimulate the tissue directly. This will reduce the number of external components needed. Finally, the result of this study opens up the way for novel stimulator designs that employ a high frequency output. In e.g., van Dongen and Serdijn (2013) a voltage steered switched-mode stimulator was proposed and simulated that also features high-power efficiency.

### Conflict of interest statement

The authors declare that the research was conducted in the absence of any commercial or financial relationships that could be construed as a potential conflict of interest.
